# The clone devaluation effect: does duplication of local facial features matter?

**DOI:** 10.1186/s13104-021-05815-1

**Published:** 2021-10-29

**Authors:** Fumiya Yonemitsu, Kyoshiro Sasaki, Akihiko Gobara, Yuki Yamada

**Affiliations:** 1 Graduate School of Humanities and Social Sciences, Hiroshima University, 1-7-1 Kagamiyama, Higashihiroshima, Hiroshima 739-8521 Japan; 2 Japan Society for the Promotion of Science, Kojimachi Business Center Building, 5-3-1 Kojimachi, Chiyoda-ku, Tokyo, 102-0083 Japan; 3 Faculty of Informatics, Kansai University, 2-1-1 Ryozenji-cho, Takatsuki, Osaka 569-1095 Japan; 4 Faculty of Arts and Science, Kyushu University, 744 Motooka, Nishi-ku, Fukuoka 819-0395 Japan; 5 BKC Research Organization of Social Sciences, Ritsumeikan University, 1-1-1 Noji-higashi, Kusatsu, Shiga 525-8577 Japan

**Keywords:** Cognition, Emotion, Face perception, Facial impression, Scrambled faces, Eeriness

## Abstract

**Objective:**

The clone devaluation is a phenomenon reported by the latest paper in which eeriness is evoked when people observe individuals with the same face (clone faces) compared to those with different faces. There are two possibilities that explain the clone devaluation effect. One is that the same facial features that clone faces have (duplication of facial features) induce the clone devaluation effect. The other possibility is that duplication of identities between people with clone faces is important for the clone devaluation effect. We thus conducted an experiment to investigate whether the duplication of identities or of facial features induces the clone devaluation effect.

**Results:**

Participants evaluated eeriness of scrambled clone faces and scrambled different faces using the paired comparison method. There was only a slight difference in subjective eeriness between scrambled clone faces and scrambled different faces. Therefore, this study suggests that the duplication of local facial features does not play a key role in inducing the clone devaluation effect.

## Introduction

**Fig. 1:**
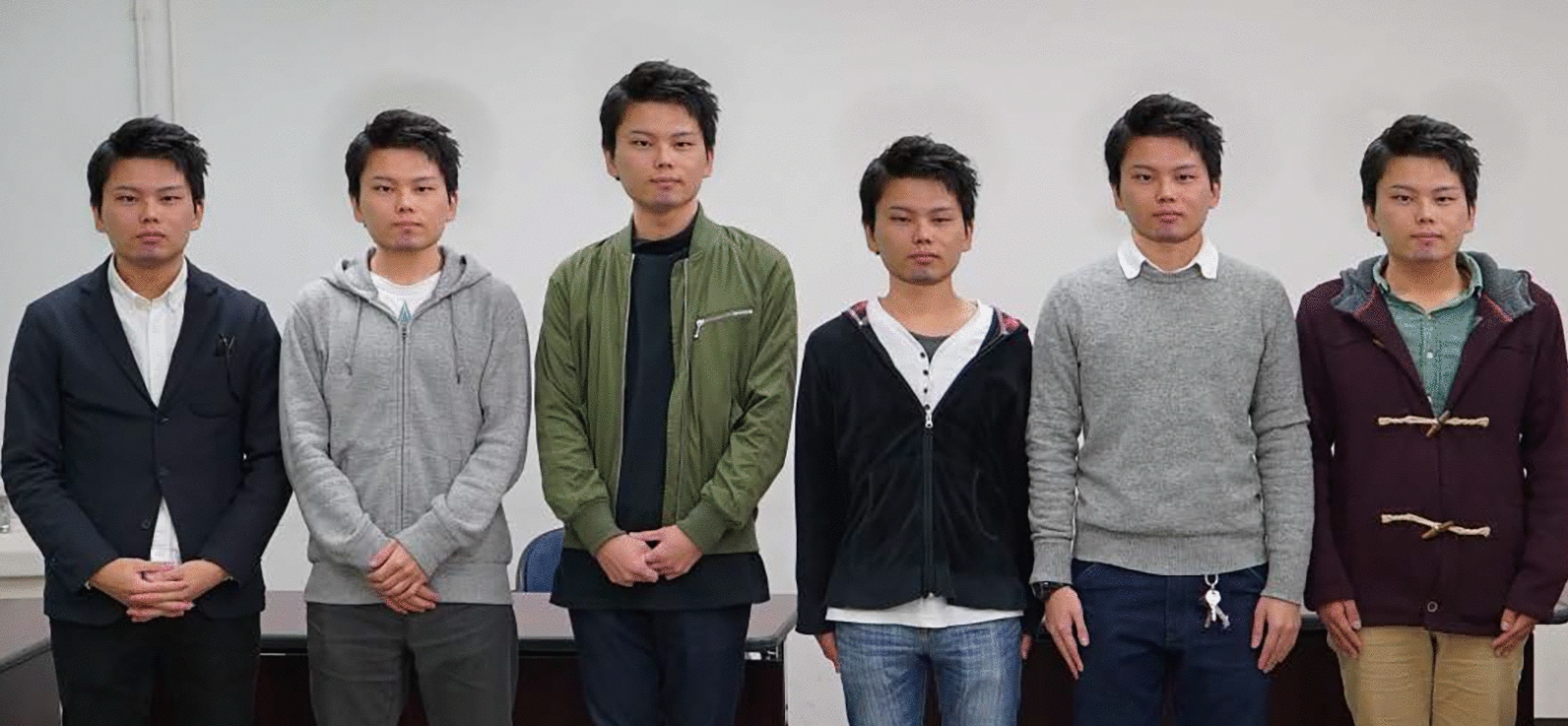
An example of clone faces

Facial impressions sometimes unexpectedly turn emotionally negative. The clone devaluation effect, identified in a recent study, is one such example [1]. In this, people who have faces with the exact same appearance (here, we call these clone faces. See also Fig. 1) elicited higher eeriness than people with different faces.

Yonemitsu et al. [1] suggested that the clone devaluation effect can be explained by two possibilities. One is that the same facial features that clone faces have (duplication of facial features) induce the clone devaluation effect. The other possibility is that the duplication of identities between people with clone faces is important for the clone devaluation effect. In Study 4a and 4b of Yonemitsu et al. [1], the authors focused on multiplets like twins as people who share almost the same facial features with each other but do not have the same identities to confirm this point. The clone faces were judged as being less eerie when presented as multiplets; however, the facial features were duplicated, supporting the latter explanations. Additionally, Yonemitsu et al. [1] reported in another experiment that clone faces of animals (e.g., dogs) did not induce the clone devaluation effect. This finding implied that having the same facial features is not always necessary to elicit the clone devaluation effect. Thus, Yonemitsu et al. [1] suggested that duplicated identities, rather than duplicated facial features, play an important role in the clone devaluation effect.

However, there are few accumulated findings on the clone devaluation effect. More evidence is needed to reveal whether duplicated identities really drive the clone devaluation effect. Therefore, the present study investigated whether duplicated identities or duplicated facial features are important to elicit the clone devaluation effect.

We manipulated faces in a way that face recognition was disrupted so that a specific person could not be identified from the face. Specifically, we used scrambled faces for clone faces and different faces. Scrambled faces are faces whose internal parts (the nose, eyes, brows, and mouth) were rearranged from the natural arrangement of facial parts [2–4]. Scrambled faces would make it difficult for people to judge whether the faces consist of clone or different faces. Thus, if the duplication of facial features induces the clone devaluation effect, scrambled clone faces would be eerier than scrambled different faces because the eeriness is evoked by the same pattern of facial features. On the other hand, if the duplication of identity induces the clone devaluation effect, there would be no significant difference in eeriness between scrambled clone faces and scrambled different faces because scrambled faces make it difficult to identify faces.

## Main text

### Methods

#### Participants

Twenty-two Japanese individuals participated in the present experiment (6 men; mean age of 21.95). All participants reported normal or corrected-to-normal vision, were fully unaware of the purpose of the experiment, and provided written, informed consent.

#### Apparatus and stimuli

**Fig. 2:**
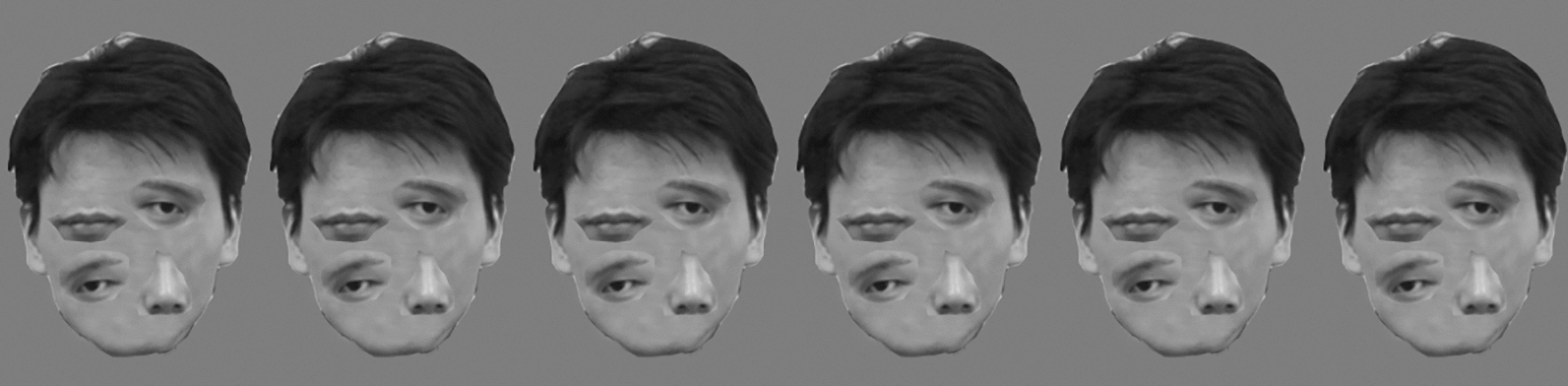
An example of scramble clone faces

We used images of six faces of Japanese males arranged in a horizontal row as stimuli (Fig. 2). There were six images of intact clone faces and six images of intact different faces. We rearranged parts of these faces to create scrambled clone and different faces. Hence, we created a total of 24 stimulus images in 4 conditions: intact clone faces, intact different faces, scrambled clone faces, and scrambled different faces. The stimuli were presented on a gamma-corrected 22-inch CRT monitor (Mitsubishi, RDF221S). The resolution of the monitor was 1024 × 768 pixels, and the refresh rate was 100 Hz. The presentation of stimuli and collection of data were controlled and performed in Matlab (The MathWorks, Natick, MA) using a computer (Apple, Mac Pro) and the Psychophysics Toolbox extension [5, 6]. It was controlled and executed by Matlab (The MathWorks, Natick, MA).

#### Procedure

The experiment was conducted in a dark room. We used Scheffe's paired comparison method. On each trial, two images were displayed: one at the top and the other at the bottom of the monitor. There were a total of 10 combinations of image conditions due to the combination of four conditions with repetition. The top and bottom positions and order of the presented images were randomized in each trial. Participants evaluated which of the images were eerier using a 5-point scale presented under the bottom image, ranging from − 2 (very eerie for the bottom image) to + 2 (very eerie for the top). There were 54 trials.

### Results

**Fig. 3:**
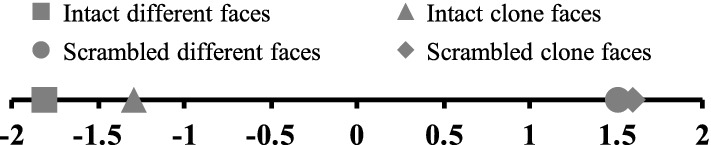
Plots of the average degree of eeriness in each condition

We calculated the average degree of eeriness for each condition (Fig. 3) and conducted multiple comparisons using Nakaya’s variant of Scheffe’s paired comparison method [7, 8]. Multiple comparisons showed that the differences between the intact different faces condition (*M* =  − 1.81) and the intact clone faces condition (*M* =  − 1.29) as well as the intact clone faces condition and the scrambled different faces condition (*M* = 1.51) were significantly larger than a yardstick (*p*s < 0.01). However, the difference between the scrambled different faces condition and scrambled clone faces condition (*M* = 1.59) was smaller than a yardstick (*p* > 0.05).

### Discussion

The experiment reported in the present study aimed at investigating whether facial features or identity play a key role in the clone devaluation effect using scrambled faces. The difference between intact faces conditions was large, while the difference between scrambled faces conditions was very small. The clone devaluation effect did not occur when clone faces were scrambled. This was possibly because face recognition was disrupted by scrambled faces, and the participants could not judge their identities as being the same. Consequently, the eeriness of the clone faces decreased even if the participants observed the clone faces. Therefore, it is suggested that the duplication of facial features is not an important factor of the clone devaluation effect. This indirectly supports the findings of Yonemitsu et al. [1].

Additionally, that intact clone faces were evaluated as being eerier than intact different faces is indicative of the occurrence of the clone devaluation effect. Yonemitsu et al. [1] used facial images, including whole bodies. On the other hand, the present study presented images in which only the face was trimmed. Despite using such stimuli, the clone devaluation effect occurred in the present study. This finding could extend the findings of Yonemitsu et al. [1]. However, the interaction between face and body in the clone devaluation effect remains unclear. Therefore, this needs to be explored by future studies.

To understand the underlying cognitive mechanisms of the clone devaluation effect, it is necessary to examine the boundary conditions under which this effect occurs in more detail in the future. In this respect, our previous study also reported that the clone devaluation effect does not occur in animal faces; this is interesting when considering it in the context of human face processing research [1]. For example, previous studies on facial categorization and visual search suggested different cognitive and neural processing between human and animal faces [9–13]. However, note that the stimuli used in our previous [1] and present studies differ in terms of the species (humans/animals) and image processing (intact/scrambled); hence, it is currently not possible to conclude whether the processing of clone faces is qualitatively or quantitatively different when the stimuli are human or animal faces. Nevertheless, the finding that the clone devaluation effect did not occur in animal faces may provide a hint for the mechanism. There are several possible explanations: (1) the identities of individual animals cannot be distinguished, (2) facial parts of animal faces cannot be processed as well as those of human faces, and (3) the existence of cloned animals does not contradict the expectation of reality. Scrambling animal faces would be useful for verification; however, we would need to develop a completely different method to do that, because no significant differences were observed in eeriness between cloned and non-cloned animal faces so far. Therefore, as many issues still need to be addressed to clarify the disappearance of the clone devaluation effect in animal faces and this is beyond the scope of this study, we will continue to examine this issue in future research.

In conclusion, the duplication of facial features does not play an important role in eliciting the clone devaluation effect, which is consistent with the findings of our previous study [1]. This claim is based on the result that the manipulation of scrambled faces yielded slight difference in eeriness between clone faces and different faces. These findings help reveal why clone devaluation effect occurs.

## Limitations

In this study, we provided evidence supporting that simple duplication of facial features is not important for inducing the clone devaluation effect. We manipulated only facial features, not identity. That is, although we may argue that the present findings are not due to facial features, we cannot strongly argue that the findings support the duplication of identity directly. Thus, our findings should be considered as indirect evidence.

The stimuli used in our experiment also suffer the limitation of ecological validity. Since the scrambled faces stimuli entailed an unusual arrangement of facial parts and were only facially trimmed, participants in the present study observed faces in a less ecologically valid context than participants in Yonemitsu et al. [1]. In fact, some previous studies imply the influence of body cues and the naturality of face stimuli on face recognition [14–19]. For example, face recognition is disrupted in an unnatural context [15]. Hence it is possible that the disruption of face recognition also occurred in the present study using unnatural facial stimuli and as a result, the difference in eeriness between the scrambled different and scrambled clone faces, which were difficult to distinguish, became negligible. The other method to disrupt face recognition is photographic negation. Unlike scrambled faces, photographic negation can disrupt face recognition while retaining the configuration of facial features [20–22]. This method is useful in investigating whether duplication of facial features is important to induce the clone devaluation effect or not, without changing the configuration. Therefore, further experiments of both manipulating identity and facial features will contribute to revealing why the clone devaluation effect occurs.

## Data Availability

The data and materials are available upon request to the first author.
